# Adiponectin attenuates high glucose-induced apoptosis through the AMPK/p38 MAPK signaling pathway in NRK-52E cells

**DOI:** 10.1371/journal.pone.0178215

**Published:** 2017-05-25

**Authors:** Yuanyuan Wang, Juan Zhang, Lian Zhang, Ping Gao, Xiaoyan Wu

**Affiliations:** Department of Nephrology, Zhongnan Hospital of Wuhan University, Wuhan, Hubei, China; University of PECS Medical School, HUNGARY

## Abstract

Excessive apoptosis of proximal tubule cell is closely related to the development of diabetes. Recent evidence suggests that adiponectin (ADPN) protects cells from high glucose induced apoptosis. However, the precise mechanisms remain poorly understood. We sought to investigate the role of p38 mitogen-activated protein kinase (p38 MAPK) and AMP activated protein kinase (AMPK) in anti-apoptotic of adiponectin under high glucose condition in rat tubular NRK-52E cells. Cells were cultured in constant and oscillating high glucose media with or without recombinant rat adiponectin for 48 h. Cell counting kit-8 (CCK-8) was used to detect cell viability, flow cytometry and Hoechst Staining were applied to investigate cell apoptosis, and western blotting was used to examine protein expression, such as phospho-AMPK and phospho-p38MAPK. Exposure to oscillating high glucose exerted lower cell viability and higher early apoptosis than constant high glucose, which were both partially prevented by adiponectin. Further studies revealed that adiponectin suppressed p38MAPK phosphorylation, but led to an increase in AMPK α phosphorylation. Compared to stable high glucose group, blockage of p38MAPK cascade with SB203580 attenuated apoptosis significantly, but failed to affect the phosphorylation level of AMPK. While AMPK inhibitor, Compound C, increased apoptosis and remarkably inhibited the p38MAPK phosphorylation. Adiponectin exert a crucial protective role against apoptosis induced by high glucose via AMPK/p38MAPK pathway.

## Introduction

Diabetes mellitus is one of the most common cause for end stage renal disease (ESRD) currently. Glomerular and vascular injuries have been regarded as the principal features of diabetic kidney diseases for years, but the effect of tubular lesions have been recognized gradually in recent year [1]. Hyperglycemia is the core initiating factor for diabetic microvascular complications, which triggers the generation of oxidant stress and free radicals in renal cells. Oscillating glucose can display more deleterious effects than stable high glucose on oxidative stress [2]. Reactive oxygen species (ROS) are précised mediators for some biological responses, such as proliferation and apoptosis [3]. Elevated glucose levels promote apoptosis in various cell lines [4–6], including tubular cells. Proximal tubular cell apoptosis is considered as one of the pathogenic mechanism of tubular atrophy and renal interstitial fibrosis, which could lead to ESRD eventually.

Many evidences indicate that the plasma level of adiponectin, an adipokine mainly secreted by adipose tissue, was decreased in diabetic patients [7]. One study in adipocytes showed that oscillating high glucose exacerbated the suppression of adiponectin mRNA expression and secretion than constant high glucose [8]. Although the protective role of adiponectin against high glucose in various cell lines has been reported [9–11], its anti-apoptotic mechanism has not been completely understood. Adiponectin exerts anti-apoptotic effect under high glucose condition in HUVECs (human umbilical vein endothelial cells) by activating AMPK [9,12], but very less research has been done in tubular cells. some reports have shown that MAPK is also involved in hyperglycemia induced apoptosis [6]. As we know, AMPK displays close relationship with p38MAPK in glucolipid metabolism [13,14], tumor metastasis [15], apoptosis [16], and so on. But their relationship in high glucose induced apoptosis has not been elucidated.

Here, we found that the effect of ADPN on high glucose caused apoptosis in NRK-52E cells and examined contributions of the AMPK-p38MAPK pathway to it.

## Materials and methods

### Cell culture and treatments

The NRK-52E cell line was purchased from the Center of Type Culture Collection (Wuhan, China), and was cultured in dulbecco modified eagle medium (DMEM, Hyclone, Logan, UT, United States) low glucose media (5.6 mM D-glucose) supplemented with 10% FBS (Sijiqing Biological Engineering Materials Co., Hangzhou, China), 100 IU/ml penicillin and 0.1 mg/ml streptomycin at 37°C under 5% CO 2 and 95% air. Cells in passages 2–3 were used. High glucose culture media were made by supplementing normal DMEM media with additional D-glucose (Sigma Chemical) to a final concentration of 30 mM. As an osmotic control, high mannitol media (HM) was made in the same way. Cells were serum restricted for 12 h, then incubated for 48h. The media were changed according to the following groups: constant normal glucose media (5.6 mM; NG), high mannitol media (NG+24.4 mM mannitol; HM), stable high glucose media (30 mM; SHG) with or without recombinant rat adiponectin (2.5μg/ml; Biovision, California, USA), intermittent high glucose media (converting from 5mM to 30 mM, back and forth per 12 h; IHG) with or without adiponectin (2.5 μg/ml). The adiponectin was added to the cell culture media, when the media was replaced, and the NRK-52E cell in adiponectin treated groups was treated with adiponectin along the whole experiment. Each group received the corresponding fresh media every 12 h.

### Assessment of cell viability

Cell viability was performed using CCK-8 (Dojindo Laboratories, Kumamoto, Japan). Cells were seeded into 96-well plates with 5 replicate wells each group at a density of 2×10^3^ cells per well with 100ul medium. After cells were incubated for indicated time, 10 ul of CCK-8 solution was added in each well for another 2 h incubation. The optical density (OD) was computed at the absorbance of 450 nm. The cell viability was calculated according to the absorbance value in each group. Results were averaged from three independently repeated experiments.

### Hoechst staining assay

Cells were seeded on slides at a density of 10 ^5^ cells/ml in 6-well plates. After reaching 70% confluence, the cells were treated as described previously for 48 h. Then Cells on slides were fixed by 4% paraformaldehyde and stained with Hoechst33258 (10μg/ml; Sigma Chemical, USA) for 10 min in sequence. Morphological changes in nuclei were observed by fluorescent microscope. Apoptotic nuclei exhibited a deep blue fluorescence, while Hoechst-negative nuclei were lightly stained blue. The relative number of positive nuclei per field (10 fields) was calculated.

### Quantification of cellular apoptosis by flow cytometry

Apoptotic cells were quantified by the Annexin-FITC Apoptosis Analysis Kit (Tianjin Sungene Biotech Co., Tianjin, China). Cells were harvested by centrifugation (300×g, 10 min). After washing by cold PBS and binding buffer in sequence, cells were resuspended by binding buffer and adjusted to a density of 10^6^ cells/ml. Annexin V-FITC (5 μl) and PI (propidium iodide, 5 μl) solutions were added successively into each tube for 10 minutes in the dark. At last, the number of apoptotic cells was analyzed by flow cytometer in 1h. The effect of SB203580 (p38 MAPK inhibitor; Sigma Chemical, USA) and Compound C (AMPK inhibitor; Sigma Chemical, USA) on apoptosis rate were detected respectively. Four independent experiments were conducted simultaneously.

### Western blot analysis

After treatment, NRK-52E cells were lysed in cell lysis solution (Beyotime, Wuhan, China) supplemented with proteinase inhibitor (Aspen, Wuhan, China) on ice for 30 min. The lysates were centrifuged at 14,000 rmp for 10 min at 4°C. The supernatants were kept and their concentrations were measured by the BCA protein assay (Beyotime, Wuhan, China). Then the protein samples were denatured at 100°C for 10 min. After being added loading buffer, equal amounts of protein were loaded in each well of 10% or 12% sodium dodecyl sulfate polyacrylamide gels and transferred onto nitrocellulose (NC) membranes at 200mA for 1.5h at 4°C. Then, the membranes were blocked with non-fat milk and incubated with the following primary antibodies overnight at 4°C: anti-p38 (Santa Cruz, 1:200 dilution), anti-p-p38 (Santa Cruz, 1:500), anti-p-AMPK α (Abcam, 1:500), anti-AMPK α (Abcam, 1:500), anti-P53 (CST, 1:1000), anti-Bax (CST, 1:1000), anti-Bcl-2 (CST, 1:1000), anti- pro caspase 3 (CST, 1:1000), anti-cleaved caspase3 (CST, 1:1000), anti-cleaved caspase 9 (CST, 1:1000), anti-pro caspase 9 (CST, 1:1000), and anti-GADPH (Santa Cruz, 1:2000). The membranes were incubated with HRP-labeled secondary antibodies (1:10000) for 1h in the following day. The luminescent signal was developed by enhanced chemical luminescence reagent (Boster, Wuhan, China) and exposed to X-ray film. The X-ray films were scanned and gray intensity analysis was quantified by Image J software. The experiment was repeated 3 times accordingly.

### Confocal laser microscope assay

After the NRK-52E cells were treated stable and intermittent high glucose with or without ADPN, the mitochondrial membrane potential was measured by confocal laser microscopy using the JC-1 Mitochondrial membrane Potential Assay Kit (Beyotime Biotechnology, Shanghai, China). Cells were grown on glass coverslips and treated with CONPs in various concentrations for 48 h. After incubating with JC-1 for 20 min, the cells were washed with staining buffer and detected by confocal laser microscopy (SP5, Leica) or flow cytometry (BD Biosciences) immediately.

### Statistical analysis

Results were presented as means ± SD. One-way ANOVA was used to analyze continuous data, following LSD post hoc tests for multiple comparisons. *P*<0.05 was considered to be statistically significant. All the above statistical analyses were performed using SPSS 17.0 software (SPSS Inc., Chicago, IL, USA).

## Results

### Adiponectin increased cell viability in NRK-52E cells exposed to high glucose

**Fig 1:**
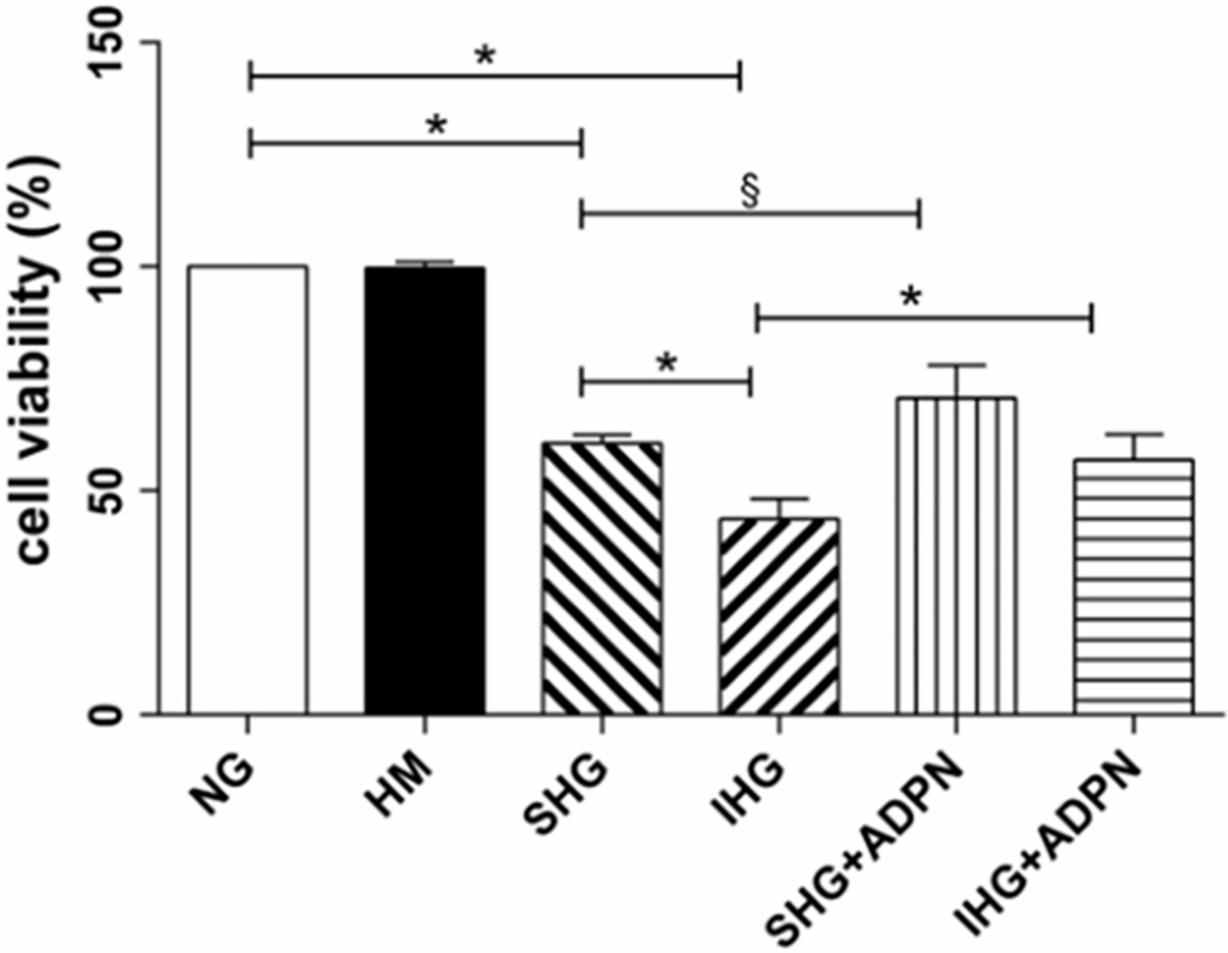
Effect of adiponectin on high glucose-induced cytotoxicty in NRK-52E cells. Cell viability was determined by CCK-8 assay. Date are shown as means±SD from three independent experiments, ^§^*P*<0.05; ^*^P<0.01. NG, normal glucose (5.6 mmol/L); HM, hyperosmotic mannital (30 mmol/L); SHG, stable high glucose (30 mmol/L); IHG, intermittent high glucose (switching from 5 to 30 mmol/L, back and forth per 12 h); SHG+ADPN, stable high glucose together with adiponectin (2.5 μg/ml); IHG+ADPN, intermittent high glucose together with adiponectin (2.5 μg/ml).

Before investigating the role of adiponectin on cell survival, we first observed the morphology of NRK-52E cells after treatment as noted previously. Before emerging cellular contact inhibition, the SHG and IHG groups showed increasing proportion of cellular hypertrophy and floating cells in culture media. Once cells reached contact inhibition, the hypertrophic morphology became inconspicuous. As shown in Fig 1, compared with normal glucose group, cell viability was significantly suppressed when high glucose was involved in, especially intermittent high glucose (*P*<0.01). However, the addition of adiponectin significantly converted cell viability in both the stable (*P*<0.05) and intermittent high glucose groups (*P*<0.01).

### Adiponectin attenuated high glucose-mediated apoptosis in NRK-52E

**Fig 2:**
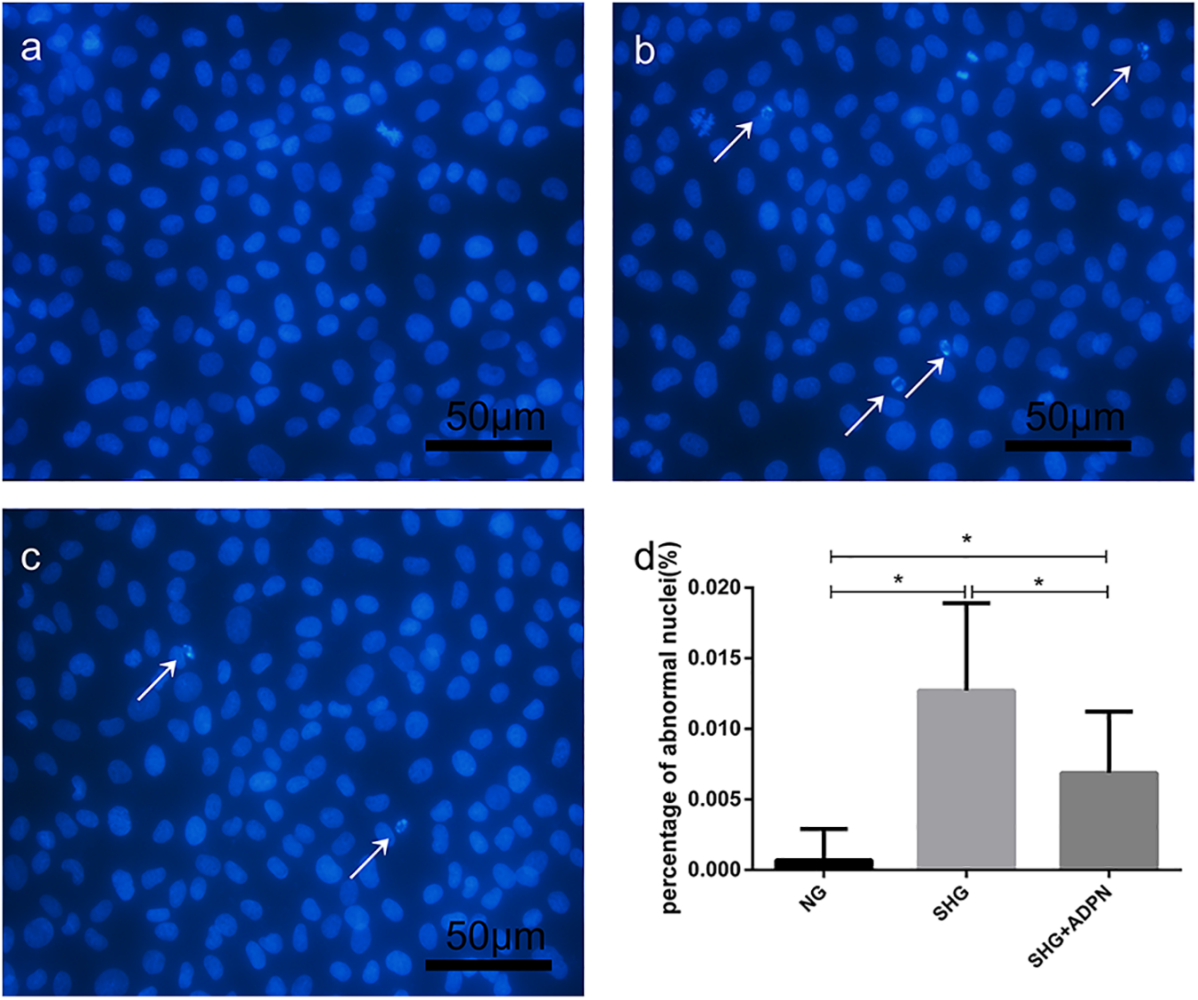
The morphologic changes in NRK-52E cells were displayed by Hoechst 33258 staining. NRK-52E cells were treated with high glucose with or without adiponectin for indicated time. Then, fluorescence images were taken after Hoechst 33258 staining. Fragmented and pycnotic nuclei were emphasized by white arrows. (200×) (a) control group. (b) SHG group. (c) SHG+ADPN group. (d) Histogram represents the percentage of apoptotic cells. ^*^P<0.01.

**Fig 3:**
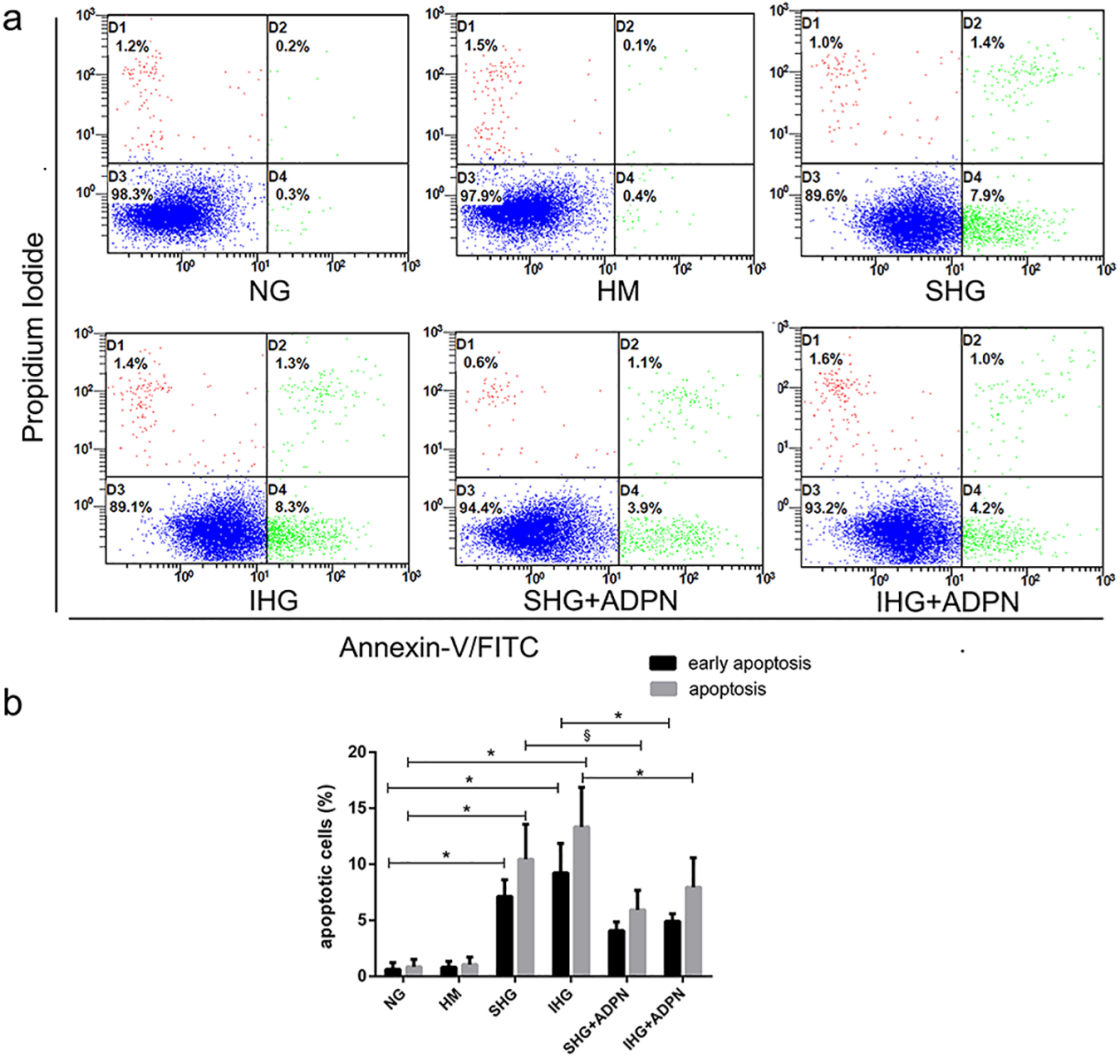
Effect of adiponectin on high glucose-induced apoptosis in NRK-52E cells. Cells were cultured with the defined concentrations of glucose in the presence or absence of adiponectin for 48 h. Flow cytometry was used to quantify cell apoptosis after Annexin V and PI double staining. (a) Flow cytometry graphical data for cell apoptosis. (b) Histogram showing percentage of early and total cell apoptosis respectively. Data are the means±SD of four independent experiments. ^§^*P*<0.05; ^*^P<0.01.

Cellular apoptosis was detected by Hoechst33258 staining. As shown in Fig 2A, nuclei were lightly stained blue in control group. In Fig 2B, we detected a significant increase in the rate of fragmented and pycnotic nuclei compared to the control group (*P*<0.01), which were deeply stained blue. But adiponectin co-treatment clearly inhibited these morphological changes of nuclei (P<0.01, Fig 2C). Furthermore, in order to examine the apoptotic rate exactly, flow cytometry, Annexin-V-FITC and PI staining were used. As displayed in Fig 3A, the early apoptosis rate of NRK-52E cells treated with stable glucose was 7.9%, 14-fold compared to the control group. And the rate for intermittent high glucose group was 8.3%. Mannitol did not exert any effect. Treatment with adiponectin at 2.5ug/ml for 48h decreased the percentage of early apoptotic cells to 3.9% and 4.2%, respectively. Fig 3B shows that there was remarkable difference in the remission for both the total and early apoptotic rate of stable high glucose group by adiponectin. And compared to intermittent high glucose group, the presence of ADPN decreased both the total and early percentage of apoptotic cells markedly, too.

### AMPK and p38MAPK are involved in the rescued effect of adiponectin on high glucose-mediated apoptosis in NRK-52E cells

**Fig 4:**
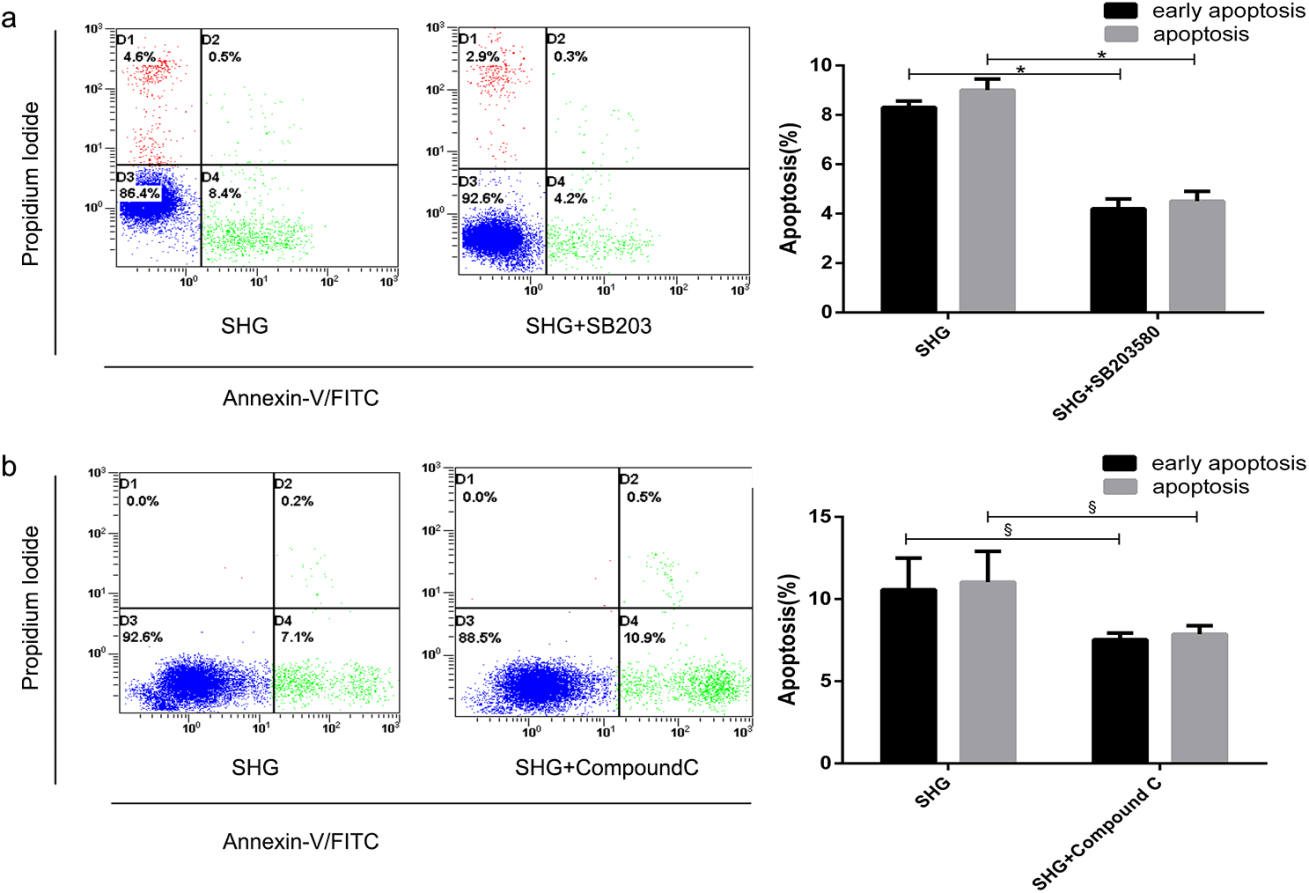
Effects of AMPK and p38MAPK inhibitor on high glucose-induced apoptosis in NRK-52E cells. The NRK-52E cells were pretreated with Compound C (10 μM) or SB203580 (10 μM) for 30min followed by incubation in high glucose media for 48 h. (a) Flow cytometry graphs for cell apoptotic rate pretreated with SB203580 and the corresponding histogram. (b) Apoptosis graphic data of NRK-52E cells pretreated with Compound C and the relevant histogram. The results are representative of three independent experiments. ^§^*P*<0.05; ^*^P<0.01.

**Fig 5:**
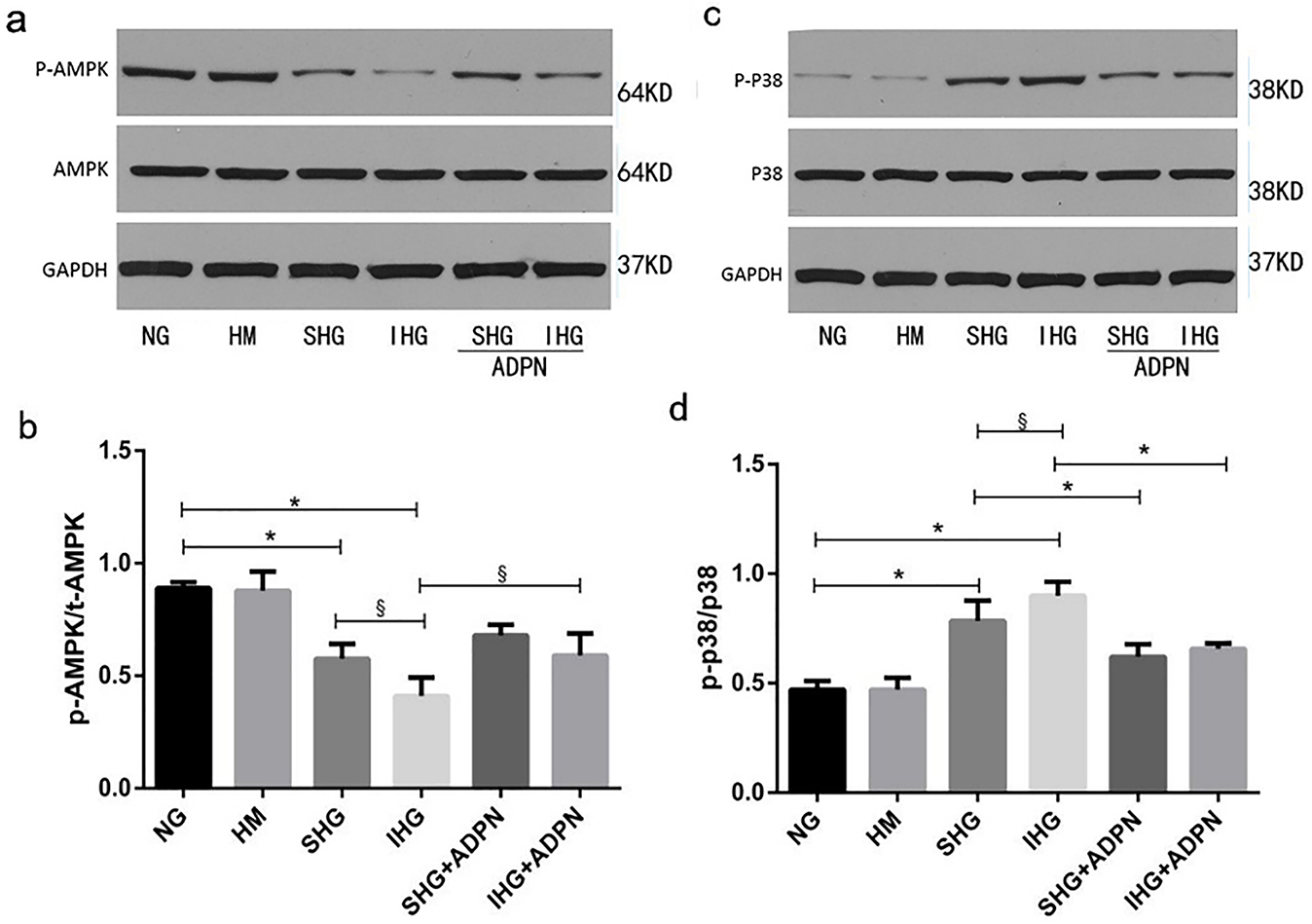
Involvement of AMPK and p38MAPK in protective effect of adiponectin against high glucose-induced apoptosis in NRK-52E cells. (a, c) Immunoblot of the phosphorylation levels of AMPK and p38MAPK in NRK-52E cells determined by total cell lysates (b, d) Histograms representing the relative levels of p-AMPK/AMPK and p-p38/p38 respectively. Data are means±SD from three independent experiments. ^§^*P*<0.05; ^*^P<0.01.

**Fig 6:**
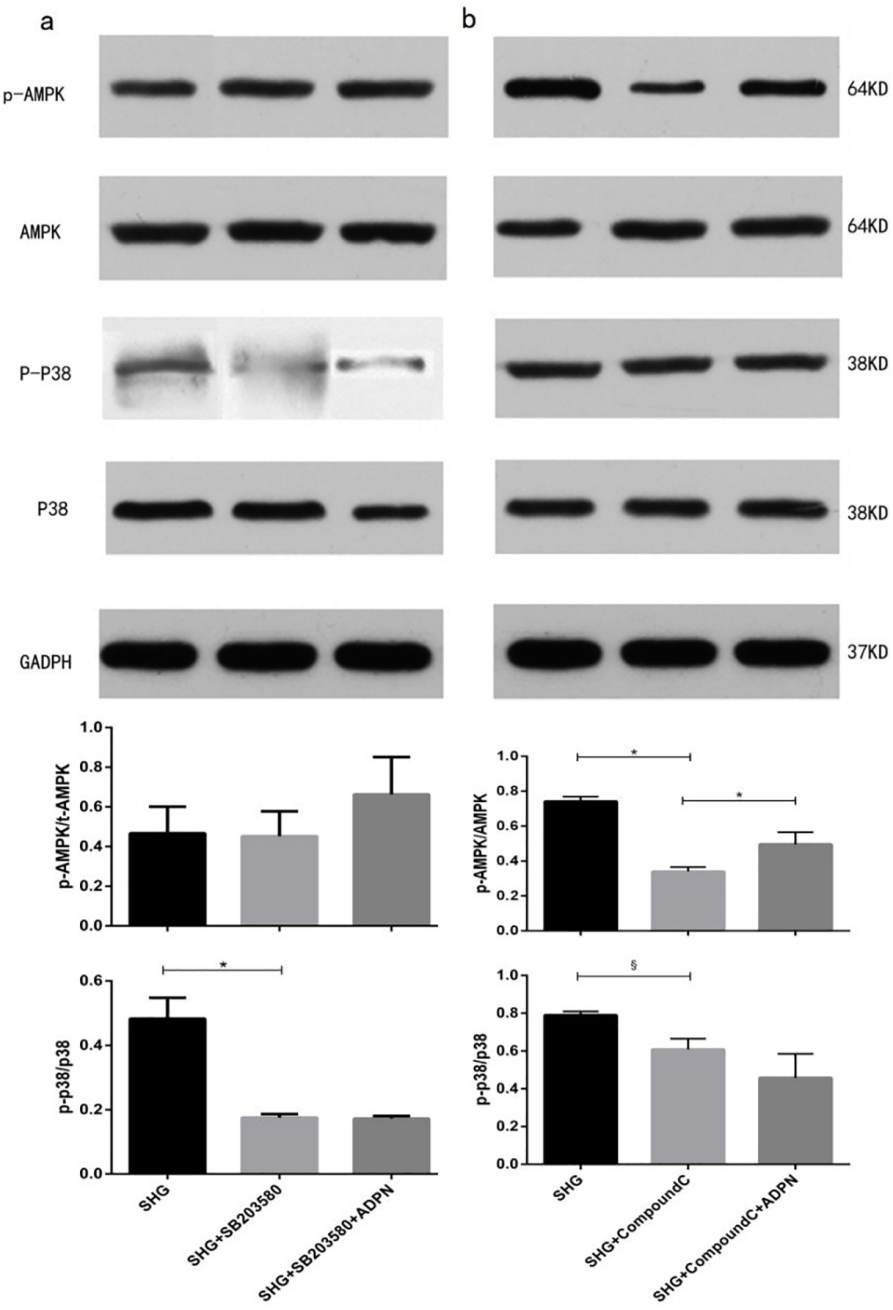
Effects of AMPK and p38MAPK inhibitors on phosphorylation of AMPK and p38MAPK in NRK-52E cells. (a) The density of p38MAPK and AMPK were determined with p38MAPK inhibitor, SB203580 and the ratios were calculated (b) p38MAPK and AMPK expression with AMPK inhibitor, Compound C, were determined and the ratios were calculated. Date are means±SD from three independent experiments. ^&^*P*<0.05;^*^P<0.01.

**Fig 7:**
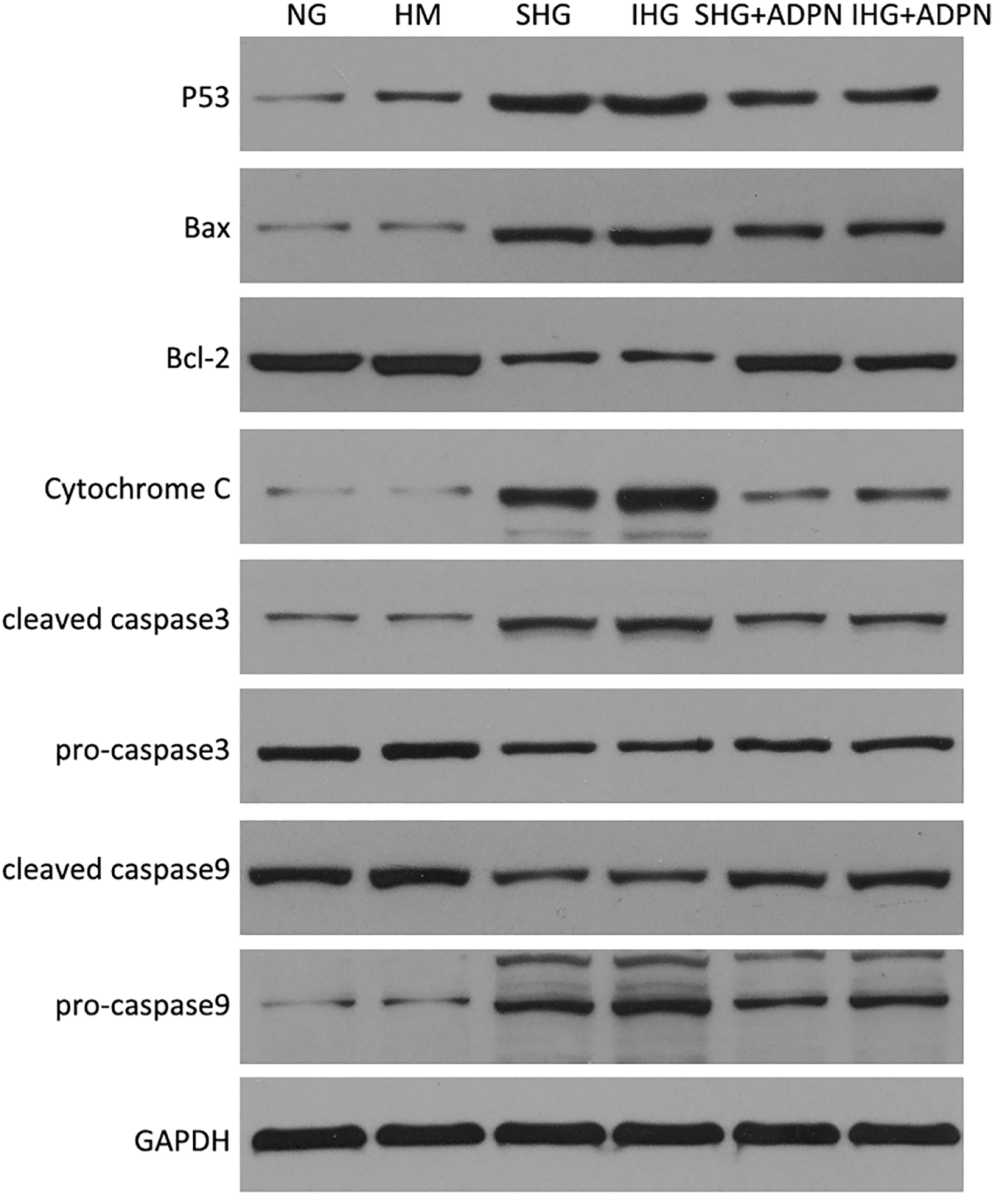
High glucose induced mitochondrion mediated apoptosis and anti-apoptosis effects of ADPN. Intermittent and stable high glucose can upregulate the protein P53, Bax. Both the intermittent and stable high glucose can downregulate the Bcl-2 protein. Both intermittent and stable high glucose induced the resealed of cytochrome C, actived the caspase 3 and caspase 9, whit the increase expression of cleaved caspase3 and caspase 9. After treated with ADPN, we found that the P53, Bax were downregulated, and the Bcl-2 was downregulated than the group without ADPN. And, after the cell treated with ADPN, the release of cytochrome C, the activation of caspase 3, caspase 9 were all inhibited.

After being treated by Compound C, the apoptosis rate was elevated significantly. (*P*<0.05 vs. SHG, Fig 4B). However, pretreated with SB203580, the apoptosis rate was attenuated (*P*<0.01 vs. SHG, Fig 4A). The level of phosphor-p38MAPK treated by high glucose was elevated (*P*<0.01 vs. NG, Fig 5D). In addition, the elevation in p38MAPK activity was more obvious in intermittent high glucose (*P*<0.05 vs. SHG group, Fig 5D). Furthermore, adiponectin partially recovered the increment of phosphor-p38MAPK in two high glucose groups (*P*<0.01 vs. SHG or IHG respectively, Fig 5D). While, the tendency of AMPK happened to be the opposite effect. Intermittent high glucose made the reduction effect on phosphorylation of AMPK worse compared to stable high glucose (*P*<0.05, Fig 5B). Adiponectin restored high glucose-mediated elevation of phospho-AMPK to a certain extent (*P*<0.01 vs. SHG or IHG respectively, Fig 5B). Compound C suppressed the high glucose-induced elevation in p38MAPK phosphorylation (*P*<0.01 vs. SHG, Fig 6B). However, SB203580 failed to change the phosphorylation level of AMPK (Fig 6A). As the P38MAPK can regulate the P53 expression, which can promote the mitochondria mediated apoptosis signaling pathway. The western blot assay showed that intermittent and stable high glucose can upregulate the protein P53, Bax, which all promotes the mitochondria mediated apoptosis. And, we also found that intermittent and stable high glucose can both downregulate the Bcl-2 protein, which inhibit the apoptosis progress. In order to test the activation of mitochondria mediated apoptosis signaling pathway, we measured the Cytochrome C, caspase 3, and caspase 9. The results showed that intermittent and stable high glucose both induced the resealed of cytochrome C, actived the caspase 3 and caspase 9, which can induce the apoptosis directly. After treated with ADPN, we found that the P53, Bax were downregulated, and the Bcl-2 was downregulated than the group without ADPN. And, after the cell treated with ADPN, the release of cytochrome C, the activation of caspase 3, caspase 9 were all inhibited, which suggested that ADPN can inhibit the mitochondria mediated apoptosis signaling pathway (Fig 7).

**Fig 8:**
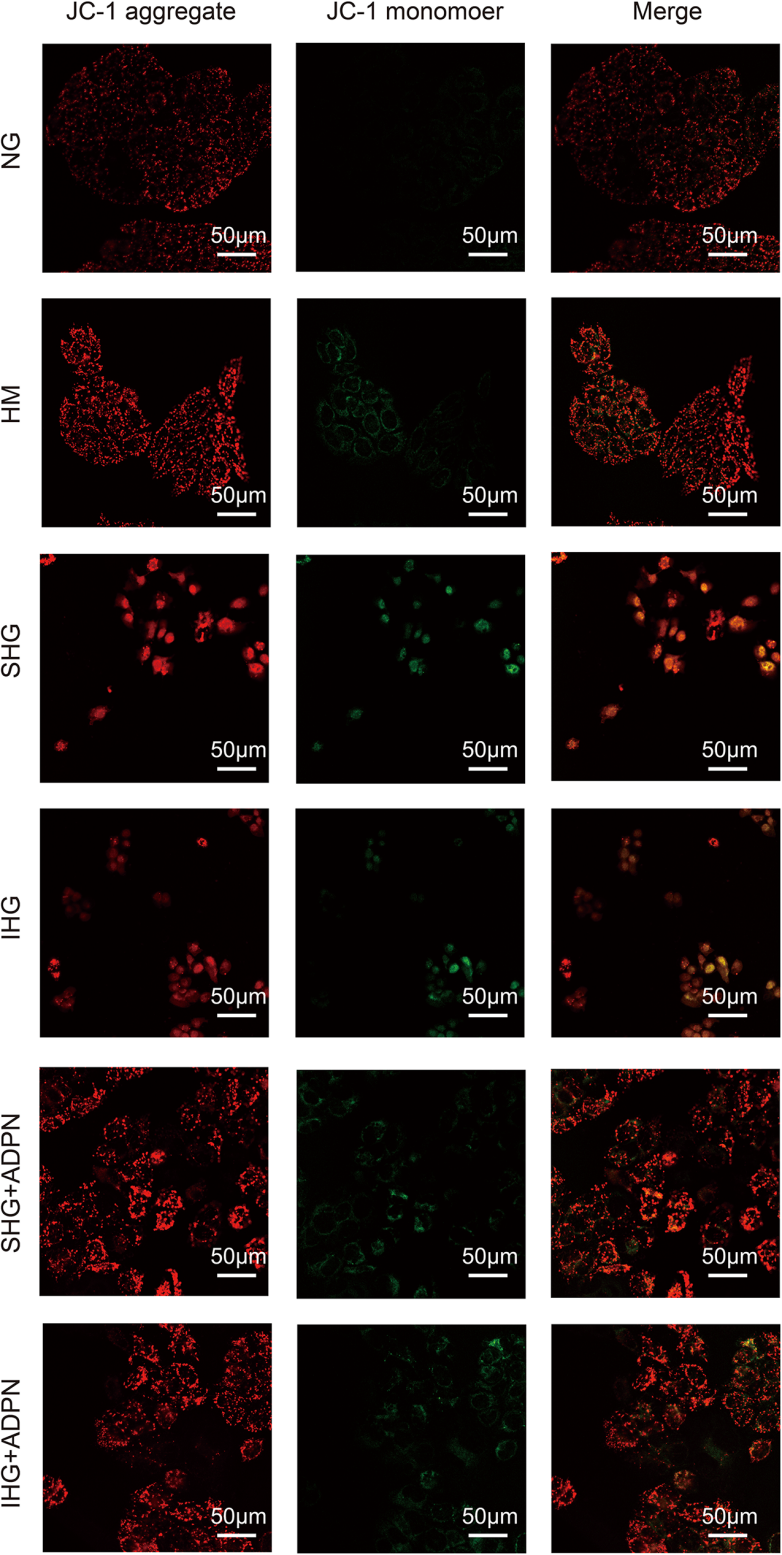
Mitochondrial membrane potential assay by confocal laser microscopy using JC-1 fluorescent dye. The intermittent and stable high glucose can downregulated the red fluorescence, and ADPN can reverse the down regulation of red fluorescence. The JC-1 in the mitochondrion is dimeride with red fluorescence, and is monomer with green fluorescence in the plasm.

As the downregulation of membrane potential of mitochondrion is an early event in the mitochondrion mediated apoptosis signaling pathway, the confocal laser microscopy assay is performed. The intermittent and stable high glucose can downregulated the mitochondrion membrane potential, and ADPN can reverse the down regulation of membrane potential (Fig 8).

## Discussion

In the present study, the rescued role of adiponectin against the damages caused by the high glucose with NRK-52E cell line as a vitro model was observed. Our study showed that adiponectin attenuated high glucose induced apoptosis by activating AMPK. p38 MAPK was a downstream target of AMPK in the anti-apoptotic effect of adiponectin.

Clinical and experimental studies strongly indicated that high glucose promotes apoptosis [9, 17]. In line with the data of Langer et al., HG was shown to induce apoptosis in renal cells [9]. Our result are in consistent with the above studies, however, the elevation of apoptosis rates induced by high glucose was not as obvious as other studies. The possible explanation is that renal tubular cells maybe more tolerant to high glucose conditions for locating in glucose re-absorption part and the media was changed every 12 h, a definite proportion of apoptosis cells were washed away with the original media. Owing to the fact that the original media were removed and the slides were washed by PBS for 3 times before Hoechst staining, the large discrepancy of apoptosis rates between Hoechst Staining and flow cytometry analysis was generated.

AMPK can serve a pro-survival as well as a pro-apoptotic effect [18]. AMPK may exert dual functions in neuronal survival, a protective role during transient energy depletion and apoptosis-inducing effect when prolonging AMPK activation [19]. The effects of ADPN on apoptosis via AMPK in kinds of cancer cell lines are in a tissue-specific fashion. Adiponectin displayed a strong anti-apoptotic role via AMPK activation in pancreatic cancer cells [20]. While, in colon cancer cells, adiponectin suppressed the proliferation and promoted apoptosis via stimulating AMPK activity, but not in human embryonic kidney (HEK293) cell line which served as a control group [21]. AMPK partially mediate the anti-apoptotic role of adiponectin on cardiovascular system [22], and associated with adiponectin anti-apoptosis effect in HUVECs [9, 12]. Our results indicated that the phosphorylation of AMPK was reduced with increased apoptosis in the high glucose medium, and when AMPK was partially weakened by adiponectin, the apoptosis was also decreased. In addition, AMPK inhibitor, Compound C could increase the apoptosis significantly in stable high glucose group. These results imply that AMPK activation drives adiponectin anti-apoptotic effect against high glucose. The effects of adiponectin on AMPK activation are dependent on different forms of adiponectin and cell lines. Although Yamauchi T et al. indicated that AMPK was activated by both full-length and globular forms of adiponectin in skeletal muscle to regulate glucose and lipid metabolism, but only by full-length adiponectin in liver [23]. However, Tsao et al. suggested that only trimeric and globular adiponectin, but not full-length form, could activate AMPK in skeletal muscle. They gave the explanation that “full-length adiponectin” Yamauchi T et al. used was not purified. And Kobayashi et al. observed that only high molecular weight form of adiponectin, but not the trimer or hexamer forms exerted antiapoptotic effect by AMPK activation in human umbilical vein endothelial cells (HUVECs) [24].

Many studies pointed out that tumor suppressor LKB1 was crucial for adiponectin-mediated AMPK activation [25]. According to one previous report, adiponectin showed a decreased ability on AMPK activation when LKB1 was in dominant negative form in myotube cells and rat hepatoma cells [26]. Moreover, adiponectin also can remarkably increase LKB1 expression and activity as well as AMPK phosphorylation in adipose stromal cells of human breast [27]. Further study is expected to clarify the upstream signaling molecules of AMPK.

Several reports have shown that p38MAPK is also involved in high glucose induced apoptosis [6]. Madhavi J.Rane et al. elucidated that the apoptosis in renal proximal tubular cells after high glucose treatment for 48 h was associated with the reduction of Akt expression followed by p38MAPK activation [28]. Junq DS et al. demonstrated that p38MAPK inhibitor, FR167653, ameliorated high glucose-stimulated mesangial cells apoptosis both in vitro and vivo condition [29]. Furthermore, some researchers found that full length adiponectin contributed to apoptosis in colon cancer SW480 cells, which may be associated with p38 activation. Our results showed that p38MAPK phosphorylation was elevated in high glucose group but relieved by adiponectin and SB203580 decreased apoptosis significantly in stable high glucose group. p38 MAPK can serve dual functions for apoptosis depending on different upstream regulator [30]. While, according to a previous report, AMPK displayed an anti-apoptotic role in human prostate cancer cells through activating P38 phosphorylation [31], which is consistent with our result. In our study the expression of p38MAPK phosphorylation was affected by Compound C, while SB203580 failed to influence AMPK phosphorylation, which implys that AMPK is the upstream regulator of p38MAPK. But the fluctuations of AMPK and p38MAPK are not coordinating. We hypothesised that other signaling pathways were involved in. Like our hypothesis, Zhang, Y et al. found that the anti-apoptotic effect of adiponectin in AMPK dominant negative mice was partially decreasing, but not disappearing [32]. Even though we confirmed that p38MAPK was a downstream target of AMPK, we could not conclude that the regulation was taken place by direct phosphorylation. Lanna, A et al. suggested that AMPK triggered recruitment of p38MAPK to transforming growth factor TGFβ-activated protein kinase-1-binging protein 1 (TAB1) in human T cells [33]. While, TAB1 can stimulates p38MAPK auto-phosphorylation. At the same time, P53 is the downstream of P38MAPK, which can promote the mitochondrion mediated apoptosis signaling pathway. So, we performed the Western blot to assay the key proteins in the mitochondrion mediated apoptosis signaling pathway, including P53, Bax, Bcl-2, Cytochrome C, caspase 3 and caspase 9. P53 can active the Bax, which can promote the apoptosis. ADPN can inhibit the P53 and Bax, and also increase the expression of Bcl-2, which can inhibit the apoptosis. ADPN can inhibit the activation of Caspase 3 and Caspase 9. And, ADPN can reverse the downregulation of mitochondrion membrane potential by high glucose, which suggested that ADPN can inhibit the mitochondrion apoptosis through AMPK/p38MAPK mediated signaling pathway.

## Conclusions

In conclusion, current results showed that both stable and fluctuating high glucose stimulated apoptosis, but fluctuating high glucose appeared to worsen the effect in NRK-52E cells. Moreover, adiponectin might have played a salutary role in suppressing apoptosis induced by high glucose through the AMPK/p38MAPK signaling pathway. Our research suggested that adiponectin show potential pharmacological effects in the diabetic nephropathy therapy after further research.
