# Correction: Adiponectin attenuates high glucose-induced apoptosis through the AMPK/p38 MAPK signaling pathway in NRK-52E cells

**DOI:** 10.1371/journal.pone.0182595

**Published:** 2017-07-27

**Authors:** Yuanyuan Wang, Juan Zhang, Lian Zhang, Ping Gao, Xiaoyan Wu

In the Funding section, the grant number from the funder National Natural Science Foundation of China is incorrect. The correct grant number is: 81170679.
